# Approximate Bayesian Computation

**DOI:** 10.1371/journal.pcbi.1002803

**Published:** 2013-01-10

**Authors:** Mikael Sunnåker, Alberto Giovanni Busetto, Elina Numminen, Jukka Corander, Matthieu Foll, Christophe Dessimoz

**Affiliations:** 1 Department of Biosystems Science and Engineering, ETH Zurich, Zurich, Switzerland; 2 Competence Center for Systems Physiology and Metabolic Diseases, Zurich, Switzerland; 3 Department of Computer Science, ETH Zurich, Zurich, Switzerland; 4 Department of Mathematics and Statistics, University of Helsinki, Helsinki, Finland; 5 CMPG Institute of Ecology and Evolution, University of Bern, Bern, Switzerland; 6 Swiss Institute of Bioinformatics, Zurich, Switzerland; 7 EMBL-European Bioinformatics Institute, Cambridge, United Kingdom; University of Toronto, Canada

## Abstract

Approximate Bayesian computation (ABC) constitutes a class of computational methods rooted in Bayesian statistics. In all model-based statistical inference, the likelihood function is of central importance, since it expresses the probability of the observed data under a particular statistical model, and thus quantifies the support data lend to particular values of parameters and to choices among different models. For simple models, an analytical formula for the likelihood function can typically be derived. However, for more complex models, an analytical formula might be elusive or the likelihood function might be computationally very costly to evaluate. ABC methods bypass the evaluation of the likelihood function. In this way, ABC methods widen the realm of models for which statistical inference can be considered. ABC methods are mathematically well-founded, but they inevitably make assumptions and approximations whose impact needs to be carefully assessed. Furthermore, the wider application domain of ABC exacerbates the challenges of parameter estimation and model selection. ABC has rapidly gained popularity over the last years and in particular for the analysis of complex problems arising in biological sciences (e.g., in population genetics, ecology, epidemiology, and systems biology).

This is a “Topic Page” article for *PLOS Computational Biology*.

## History

The first Approximate Bayesian computation (ABC)-related ideas date back to the 1980s. Donald Rubin, when discussing the interpretation of Bayesian statements in 1984 [1], described a hypothetical sampling mechanism that yields a sample from the posterior distribution. This scheme was more of a conceptual thought experiment to demonstrate what type of manipulations are done when inferring the posterior distributions of parameters. The description of the sampling mechanism coincides exactly with that of the ABC-rejection scheme, and this article can be considered to be the first to describe approximate Bayesian computation. Another prescient point was made when Rubin argued that in Bayesian inference, applied statisticians should not settle for analytically tractable models only but instead consider computational methods that allow them to estimate the posterior distribution of interest. This way, a wider range of models can be considered. These arguments are particularly relevant in the context of ABC.

In 1984, Peter Diggle and Richard Gratton suggested using a systematic simulation scheme to approximate the likelihood function in situations where its analytic form is intractable [2]. Their method was based on defining a grid in the parameter space and using it to approximate the likelihood by running several simulations for each grid point. The approximation was then improved by applying smoothing techniques to the outcomes of the simulations. While the idea of using simulation for hypothesis testing was not new [3], [4], Diggle and Gratton seemingly introduced the first procedure using simulation to do statistical inference under a circumstance where the likelihood is intractable.

Although Diggle and Gratton's approach had opened a new frontier, their method was not yet exactly identical to what is now known as ABC, as it aimed at approximating the likelihood rather than the posterior distribution. An article of Simon Tavaré et al. [5] was first to propose an ABC algorithm for posterior inference. In their seminal work, inference about the genealogy of DNA sequence data was considered, and in particular the problem of deciding the posterior distribution of the time to the most recent common ancestor of the sampled individuals. Such inference is analytically intractable for many demographical models, but the authors presented ways of simulating coalescent trees under the putative models. A sample from the posterior of model parameters was obtained by accepting/rejecting proposals based on comparing the number of segregating sites in the synthetic and real data. This work was followed by an applied study on modeling the variation in human Y chromosome by Jonathan K. Pritchard et al. [6] using the ABC method. Finally, the term Approximate Bayesian Computation was established by Mark Beaumont et al. [7], extending further the ABC methodology and discussing the suitability of the ABC-approach more specifically for problems in population genetics. Since then, ABC has spread to applications outside population genetics, such as systems biology, epidemiology, or phylogeography.

## Method

### Motivation

A common incarnation of the Bayes theorem relates the conditional probability (or density) of a particular parameter value 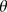 given data 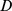 to the probability of 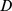 given 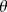 by the rule:
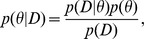
where 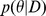 denotes the posterior, 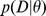 the likelihood, 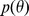 the prior, and 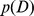 the evidence (also referred to as the marginal likelihood or the prior predictive probability of the data).

The prior represents beliefs about 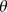 before 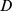 is available, and it is often specified by choosing a particular distribution among a set of well-known and tractable families of distributions, such that both the evaluation of prior probabilities and random generation of values of 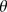 are relatively straightforward. For certain kinds of models, it is more pragmatic to specify the prior 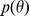 using a factorization of the joint distribution of all the elements of 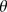 in terms of a sequence of their conditional distributions. If one is only interested in the relative posterior plausibilities of different values of 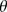, the evidence 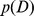 can be ignored, as it constitutes a normalising constant, which cancels for any ratio of posterior probabilities. It remains, however, necessary to evaluate the likelihood 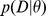 and the prior 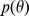. For numerous applications, it is computationally expensive, or even completely infeasible, to evaluate the likelihood [8], which motivates the use of ABC to circumvent this issue.

### The ABC Rejection Algorithm

**Figure 1:**
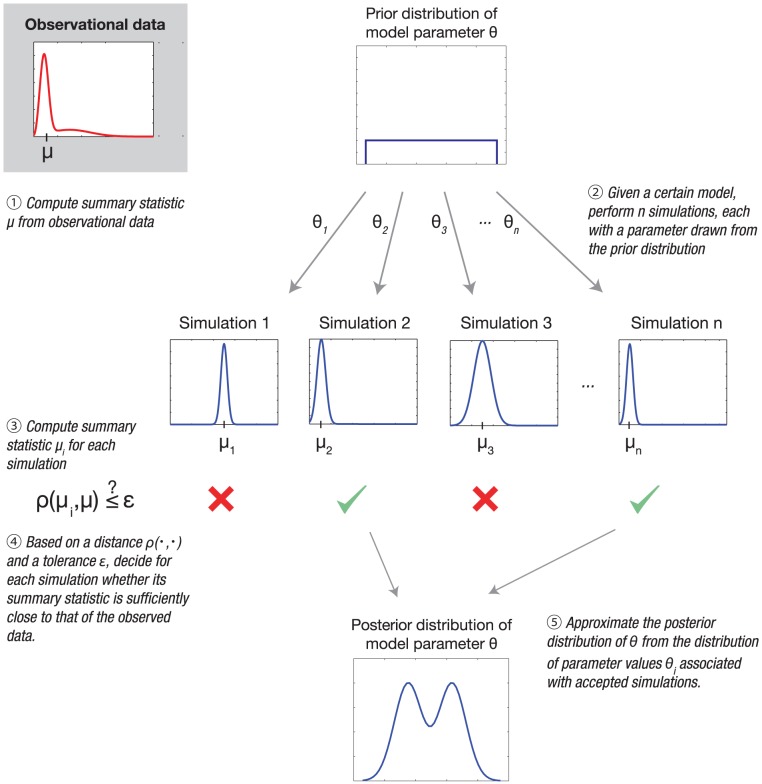
Parameter estimation by Approximate Bayesian Computation: a conceptual overview.

All ABC-based methods approximate the likelihood function by simulations, the outcomes of which are compared with the observed data [9]–[11]. More specifically, with the ABC rejection algorithm—the most basic form of ABC—a set of parameter points is first sampled from the prior distribution. Given a sampled parameter point 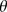, a dataset 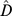 is then simulated under the statistical model 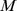 specified by 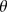. If the generated 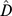 is too different from the observed data 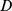, the sampled parameter value is discarded. In precise terms, 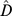 is accepted with tolerance 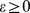 if:
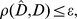
where the distance measure 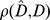 determines the level of discrepancy between  and 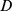 based on a given metric (e.g., the Euclidean distance). A strictly positive tolerance is usually necessary, since the probability that the simulation outcome coincides exactly with the data (event 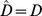) is negligible for all but trivial applications of ABC, which would in practice lead to rejection of nearly all sampled parameter points. The outcome of the ABC rejection algorithm is a sample of parameter values approximately distributed according to the desired posterior distribution and, crucially, obtained without the need of explicitly evaluating the likelihood function (Figure 1).

### Summary Statistics

The probability of generating a dataset 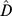 with a small distance to 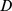 typically decreases as the dimensionality of the data increases. This leads to a substantial decrease in the computational efficiency of the above basic ABC rejection algorithm. A common approach to lessen this problem is to replace 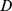 with a set of lower dimensional summary statistics 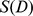, which are selected to capture the relevant information in 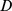. The acceptance criterion in ABC rejection algorithm becomes:
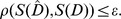



If the summary statistics are sufficient with respect to the model parameters 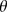, the efficiency increase obtained in this way does not introduce any error [12]. Indeed, by definition, sufficiency implies that all information in 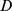 about 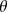 is captured by 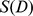.

As elaborated below, it is typically impossible, outside the exponential family of distributions, to identify a finite-dimensional set of sufficient statistics. Nevertheless, informative, but possibly nonsufficient, summary statistics are often used in applications where inference is performed with ABC methods.

## Example

**Figure 2:**
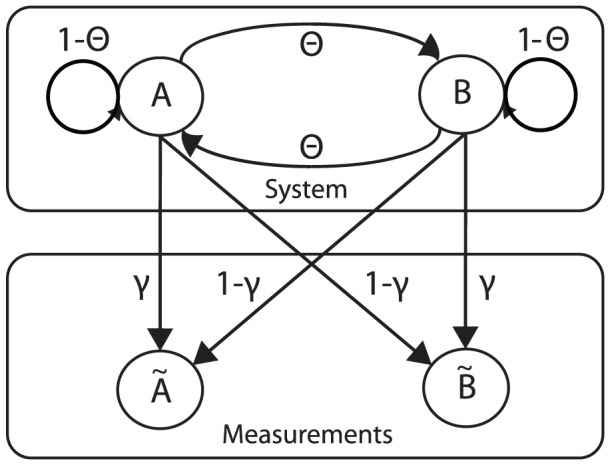
A dynamic bistable hidden Markov model.

An illustrative example is a bistable system that can be characterized by a hidden Markov model (HMM) subject to measurement noise (Figure 2). Such models are employed for many biological systems: they have for example been used in Development, signaling, activation/deactivation, logical processing, and non-equilibrium thermodynamics. For instance, the behavior of the Sonic Hedgehog (Shh) transcription factor in Drosophila melanogaster can be modeled with a HMM [13]. The (biological) dynamical model consists of two states:  and 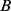. If the probability of a transition from one state to the other is defined as 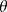 in both directions, the probability to remain in the same state at each time step is 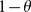. The probability to measure the state correctly is 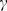 (conversely, the probability of an incorrect measurement is 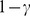).

Due to the conditional dependencies between states at different time points, calculation of the likelihood of time series data is somewhat tedious, which illustrates the motivation to use ABC. A computational issue for the basic ABC is the large dimensionality of the data in an application like this. This can be reduced using the summary statistic 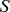, which is the frequency of switches between the two states. As a distance measure 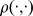, the absolute difference is used, combined with a tolerance 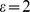. The posterior inference about the parameter 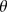 can be done following the five steps presented in Figure 1:

**Step 1:** Assume that the observed data are the state sequence AAAABAABBAAAAAABAAAA, which was generated using 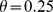. The associated summary statistic, the number of switches between the states in the experimental data, is 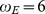.

**Step 2:** Assuming nothing is known about 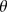, a uniform prior in the interval 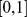 is employed. A number 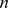 of parameter points are drawn from the prior, and the model is simulated for each of the parameter points 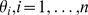, which results in 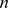 sequences of simulated data. In this example, 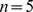, with each drawn parameter and simulated dataset recorded in Table 1, column 2–3. In practice, 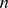 would need to be much larger to obtain an appropriate approximation.

**Table 1:** Example of ABC rejection algorithm.

i	θ*_i_*	Simulated Datasets (Step 2)	Summary Statistic ω*_S_*_,*i*_ (Step 3)	Distance ρ (ω*_S_*_,*i*_,ω*_E_*) (Step 4)	Outcome (Step 4)
1	0.08	AABAAAABAABAAABAAAAA	8	2	accepted
2	0.68	AABBABABAAABBABABBAB	13	7	rejected
3	0.87	BBBABBABBBBABABBBBBA	9	3	rejected
4	0.43	AABAAAAABBABBBBBBBBA	6	0	accepted
5	0.53	ABBBBBAABBABBABAABBB	9	3	rejected

**Step 3:** The summary statistic is being computed for each sequence of simulated data, 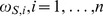 (Table 1, column 4).

**Step 4:** The distance between the observed and simulated transition frequencies 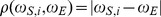 is computed for all parameter points (Table 1, column 5). Parameter points for which the distance is smaller than or equal to 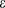 are accepted as approximate samples from the posterior (Table 1, column 6).

**Step 5:** The posterior distribution is approximated with the accepted parameter points. The posterior distribution should have a nonnegligible probability for parameter values in a region around the true value of 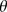 in the system, if the data are sufficiently informative. In this example, the posterior probability mass is evenly split between the values 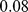 and 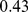.

**Figure 3:**
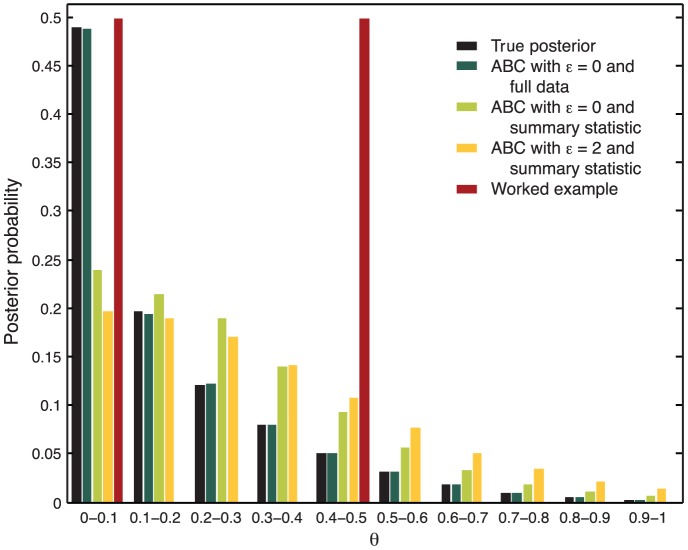
Posterior of θ obtained in the example (red), compared with the true posterior distribution (black), and ABC simulations with large *n*. The use of the insufficient summary statistic ω introduces a bias, even when requiring ε = 0 (light green).

Figure 3 shows the posterior probabilities obtained by ABC and a large 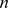 using either the summary statistic combined with (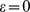 and 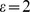) or the full data sequence. These are compared with the true posterior, which can be computed exactly and efficiently using the Viterbi algorithm. The used summary statistic is not sufficient, and it is seen that even with 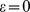, the deviation from the theoretical posterior is considerable. Of note, a much longer observed data sequence would be required to obtain a posterior that is concentrated around the true value of 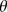 (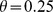).

This example application of ABC used simplifications for illustrative purposes. A number of review articles provide pointers to more realistic applications of ABC [9]–[11], [14].

## Model Comparison with ABC

Besides parameter estimation, the ABC-framework can be used to compute the posterior probabilities of different candidate models [15]–[17]. In such applications, one possibility is to use the rejection-sampling in a hierarchical manner. First, a model is sampled from the prior distribution for the models; then, given the model sampled, the model parameters are sampled from the prior distribution assigned to that model. Finally, a simulation is performed as in the single-model ABC. The relative acceptance frequencies for the different models now approximate the posterior distribution for these models. Again, computational improvements for ABC in the space of models have been proposed, such as constructing a particle filter in the joint space of models and parameters [17].

Once the posterior probabilities of models have been estimated, one can make full use of the techniques of Bayesian model comparison. For instance, to compare the relative plausibilities of two models 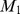 and 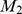, one can compute their posterior ratio, which is related to the Bayes factor 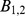:
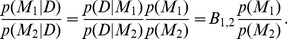



If the model priors are equal (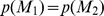), the Bayes factor equals the posterior ratio.

In practice, as discussed below, these measures can be highly sensitive to the choice of parameter prior distributions and summary statistics, and thus conclusions of model comparison should be drawn with caution.

## Pitfalls and Remedies

As for all statistical methods, a number of assumptions and approximations are inherently required for the application of ABC-based methods to real modeling problems. For example, setting the tolerance parameter 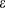 to zero ensures an exact result but typically makes computations prohibitively expensive. Thus, values of 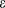 larger than zero are used in practice, which introduces a bias. Likewise, sufficient statistics are typically not available, and instead, other summary statistics are used, which introduces an additional bias due to the loss of information. Additional sources of bias—for example, in the context of model selection—may be more subtle [12], [18].

At the same time, some of the criticisms that have been directed at the ABC methods, in particular within the field of phylogeography [19]–[21], are not specific to ABC and apply to all Bayesian methods or even all statistical methods (e.g., the choice of prior distribution and parameter ranges) [9], [22]. However, because of the ability of ABC-methods to handle much more complex models, some of these general pitfalls are of particular relevance in the context of ABC analyses.

This section discusses these potential risks and reviews possible ways to address them (Table 2).

**Table 2:** Potential risks and remedies in ABC-based statistical inference.

Error Source	Potential Issue	Solution	Subsection
Nonzero tolerance ε	The inexactness introduces a bias in the computed posterior distribution.	Theoretical/practical studies of the sensitivity of the posterior distribution to the tolerance. Noisy ABC.	Approximation of the posterior
Nonsufficient summary statistics	The information loss causes inflated credible intervals.	Automatic selection/semi-automatic identification of sufficient statistics. Model validation checks (e.g., Templeton 2009 [19]).	Choice and sufficiency of summary statistics
Small number of models/mis-specified models	The investigated models are not representative/lack predictive power.	Careful selection of models. Evaluation of the predictive power.	Small number of models
Priors and parameter ranges	Conclusions may be sensitive to the choice of priors. Model choice may be meaningless.	Check sensitivity of Bayes factors to the choice of priors. Some theoretical results regarding choice of priors are available. Use alternative methods for model validation.	Prior distribution and parameter ranges
Curse-of-dimensionality	Low parameter acceptance rates. Model errors cannot be distinguished from an insufficient exploration of the parameter space. Risk of overfitting.	Methods for model reduction if applicable. Methods to speed up the parameter exploration. Quality controls to detect overfitting.	Curse-of-dimensionality
Model ranking with summary statistics	The computation of Bayes factors on summary statistics may not be related to the Bayes factors on the original data, which may therefore render the results meaningless.	Only use summary statistics that fulfill the necessary and sufficient conditions to produce a consistent Bayesian model choice. Use alternative methods for model validation.	Bayes factor with ABC and summary statistics
Implementation	Low protection to common assumptions in the simulation and the inference process.	Sanity checks of results. Standardization of software.	Indispensable quality controls

### Approximation of the Posterior

A nonnegligible 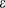 comes with the price that one samples from 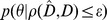 instead of the true posterior 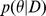. With a sufficiently small tolerance, and a sensible distance measure, the resulting distribution 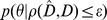 should often approximate the actual target distribution 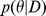 reasonably well. On the other hand, a tolerance that is large enough that every point in the parameter space becomes accepted will yield a replica of the prior distribution. There are empirical studies of the difference between 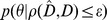 and 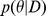 as a function of 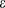 [23], and theoretical results for an upper 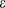-dependent bound for the error in parameter estimates [24]. The accuracy of the posterior (defined as the expected quadratic loss) delivered by ABC as a function of 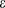 has also been investigated [25]. However, the convergence of the distributions when 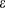 approaches zero, and how it depends on the distance measure used, is an important topic that has yet to be investigated in greater detail. In particular, it remains difficult to disentangle errors introduced by this approximation from errors due to model mis-specification [9].

As an attempt to correct some of the error due to a non-zero 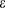, the usage of local linear weighted regression with ABC to reduce the variance of the posterior estimates has been suggested [7]. The method assigns weights to the parameters according to how well simulated summaries adhere to the observed ones and performs linear regression between the summaries and the weighted parameters in the vicinity of observed summaries. The obtained regression coefficients are used to correct sampled parameters in the direction of observed summaries. An improvement was suggested in the form of nonlinear regression using a feed-forward neural network model [26]. However, it has been shown that the posterior distributions obtained with these approaches are not always consistent with the prior distribution, which did lead to a reformulation of the regression adjustment that respects the prior distribution [27].

Finally, statistical inference using ABC with a non-zero tolerance 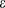 is not inherently flawed: under the assumption of measurement errors, the optimal 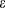 can in fact be shown to be not zero [25], [28]. Indeed, the bias caused by a non-zero tolerance can be characterized and compensated by introducing a specific form of noise to the summary statistics. Asymptotic consistency for such “noisy ABC” has been established, together with formulas for the asymptotic variance of the parameter estimates for a fixed tolerance [25].

### Choice and Sufficiency of Summary Statistics

Summary statistics may be used to increase the acceptance rate of ABC for high-dimensional data. Low-dimensional sufficient statistics are optimal for this purpose, as they capture all relevant information present in the data in the simplest possible form [11]. However, low-dimensional sufficient statistics are typically unattainable for statistical models where ABC-based inference is most relevant, and consequently, some heuristic is usually necessary to identify useful low-dimensional summary statistics. The use of a set of poorly chosen summary statistics will often lead to inflated credible intervals due to the implied loss of information [11], which can also bias the discrimination between models. A review of methods for choosing summary statistics is available [29], which may provide valuable guidance in practice.

One approach to capture most of the information present in data would be to use many statistics, but the accuracy and stability of ABC appears to decrease rapidly with an increasing numbers of summary statistics [9], [11]. Instead, a better strategy is to focus on the relevant statistics only—relevancy depending on the whole inference problem, on the model used, and on the data at hand [30].

An algorithm has been proposed for identifying a representative subset of summary statistics, by iteratively assessing whether an additional statistic introduces a meaningful modification of the posterior [31]. One of the challenges here is that a large ABC approximation error may heavily influence the conclusions about the usefulness of a statistic at any stage of the procedure. Another method decomposes into two main steps [30]. First, a reference approximation of the posterior is constructed by minimizing the entropy. Sets of candidate summaries are then evaluated by comparing the ABC-approximated posteriors with the reference posterior.

With both of these strategies, a subset of statistics is selected from a large set of candidate statistics. Instead, the partial least squares regression approach uses information from all the candidate statistics, each being weighted appropriately [32]. Recently, a method for constructing summaries in a semi-automatic manner has attained a considerable interest [25]. This method is based on the observation that the optimal choice of summary statistics, when minimizing the quadratic loss of the parameter point estimates, can be obtained through the posterior mean of the parameters, which is approximated by performing a linear regression based on the simulated data.

Methods for the identification of summary statistics that could also simultaneously assess the influence on the approximation of the posterior would be of substantial value [33]. This is because the choice of summary statistics and the choice of tolerance constitute two sources of error in the resulting posterior distribution. These errors may corrupt the ranking of models and may also lead to incorrect model predictions. Indeed, none of the methods above assess the choice of summaries for the purpose of model selection.

### Bayes Factor with ABC and Summary Statistics

It has been shown that the combination of insufficient summary statistics and ABC for model selection can be problematic [12], [18]. Indeed, if one lets the Bayes factor based on the summary statistic 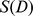 be denoted by 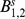, the relation between 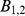 and 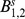 takes the form [12]:
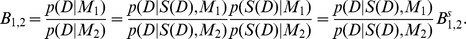



Thus, a summary statistic 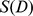 is sufficient for comparing two models 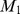 and 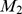 if and only if:
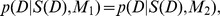
which results in that 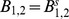. It is also clear from the equation above that there might be a huge difference between 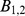 and 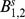 if the condition is not satisfied, as can be demonstrated by toy examples [12], [16], [18]. Crucially, it was shown that sufficiency for 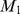 or 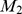 alone, or for both models, does not guarantee sufficiency for ranking the models [12]. However, it was also shown that any sufficient summary statistic for a model 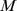 in which both 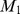 and 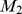 are nested is valid for ranking the nested models [12].

The computation of Bayes factors on 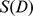 may therefore be misleading for model selection purposes, unless the ratio between the Bayes factors on 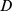 and 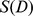 would be available, or at least could be approximated reasonably well. Alternatively, necessary and sufficient conditions on summary statistics for a consistent Bayesian model choice have recently been derived [34], which can provide useful guidance.

However, this issue is only relevant for model selection when the dimension of the data has been reduced. ABC-based inference, in which the actual datasets are directly compared—as is the case for some systems biology applications (e.g., see [35])—circumvents this problem.

### Indispensable Quality Controls

As the above discussion makes clear, any ABC analysis requires choices and tradeoffs that can have a considerable impact on its outcomes. Specifically, the choice of competing models/hypotheses, the number of simulations, the choice of summary statistics, or the acceptance threshold cannot currently be based on general rules, but the effect of these choices should be evaluated and tested in each study [10].

A number of heuristic approaches to the quality control of ABC have been proposed, such as the quantification of the fraction of parameter variance explained by the summary statistics [10]. A common class of methods aims at assessing whether or not the inference yields valid results, regardless of the actually observed data. For instance, given a set of parameter values, which are typically drawn from the prior or the posterior distributions for a model, one can generate a large number of artificial datasets. In this way, the quality and robustness of ABC inference can be assessed in a controlled setting, by gauging how well the chosen ABC inference method recovers the true parameter values, and also models if multiple structurally different models are considered simultaneously.

Another class of methods assesses whether the inference was successful in light of the given observed data, for example by comparing the posterior predictive distribution of summary statistics to the summary statistics observed [10]. Beyond that, cross-validation techniques [36] and predictive checks [37], [38] represent promising future strategies to evaluate the stability and out-of-sample predictive validity of ABC inferences. This is particularly important when modeling large datasets, because then the posterior support of a particular model can appear overwhelmingly conclusive, even if all proposed models in fact are poor representations of the stochastic system underlying the observation data. Out-of-sample predictive checks can reveal potential systematic biases within a model and provide clues on to how to improve its structure or parametrization.

Interestingly, fundamentally novel approaches for model choice that incorporate quality control as an integral step in the process have recently been proposed. ABC allows, by construction, estimation of the discrepancies between the observed data and the model predictions, with respect to a comprehensive set of statistics. These statistics are not necessarily the same as those used in the acceptance criterion. The resulting discrepancy distributions have been used for selecting models that are in agreement with many aspects of the data simultaneously [39], and model inconsistency is detected from conflicting and codependent summaries. Another quality-control-based method for model selection employs ABC to approximate the effective number of model parameters and the deviance of the posterior predictive distributions of summaries and parameters [40]. The deviance information criterion is then used as measure of model fit. It has also been shown that the models preferred based on this criterion can conflict with those supported by Bayes factors. For this reason, it is useful to combine different methods for model selection to obtain correct conclusions.

Quality controls are achievable and indeed performed in many ABC-based works, but for certain problems, the assessment of the impact of the method-related parameters can be challenging. However, the rapidly increasing use of ABC can be expected to provide a more thorough understanding of the limitations and applicability of the method.

### General Risks in Statistical Inference Exacerbated in ABC

This section reviews risks that are strictly speaking not specific to ABC, but also relevant for other statistical methods as well. However, the flexibility offered by ABC to analyze very complex models makes them highly relevant to discuss here.

#### Prior distribution and parameter ranges

The specification of the range and the prior distribution of parameters strongly benefits from previous knowledge about the properties of the system. One criticism has been that in some studies the “parameter ranges and distributions are only guessed based upon the subjective opinion of the investigators” [41], which is connected to classical objections of Bayesian approaches [42].

With any computational method, it is typically necessary to constrain the investigated parameter ranges. The parameter ranges should if possible be defined based on known properties of the studied system but may for practical applications necessitate an educated guess. However, theoretical results regarding objective priors are available, which may for example be based on the principle of indifference or the principle of maximum entropy [43], [44]. On the other hand, automated or semi-automated methods for choosing a prior distribution often yield improper densities. As most ABC procedures require generating samples from the prior, improper priors are not directly applicable to ABC.

One should also keep the purpose of the analysis in mind when choosing the prior distribution. In principle, uninformative and flat priors that exaggerate our subjective ignorance about the parameters may still yield reasonable parameter estimates. However, Bayes factors are highly sensitive to the prior distribution of parameters. Conclusions on model choice based on Bayes factor can be misleading unless the sensitivity of conclusions to the choice of priors is carefully considered.

#### Small number of models

Model-based methods have been criticized for not exhaustively covering the hypothesis space [21]. Indeed, model-based studies often revolve around a small number of models, and due to the high computational cost to evaluate a single model in some instances, it may then be difficult to cover a large part of the hypothesis space.

An upper limit to the number of considered candidate models is typically set by the substantial effort required to define the models and to choose between many alternative options [10]. There is no commonly accepted ABC-specific procedure for model construction, so experience and prior knowledge are used instead [11]. Although more robust procedures for a priori model choice and formulation would be beneficial, there is no one-size-fits-all strategy for model development in statistics: sensible characterization of complex systems will always necessitate a great deal of detective work and use of expert knowledge from the problem domain.

Some opponents of ABC contend that since only few models—subjectively chosen and probably all wrong—can be realistically considered, ABC analyses provide only limited insight [21]. However, there is an important distinction between identifying a plausible null hypothesis and assessing the relative fit of alternative hypotheses [9]. Since useful null hypotheses, that potentially hold true, can extremely seldom be put forward in the context of complex models, predictive ability of statistical models as explanations of complex phenomena is far more important than the test of a statistical null hypothesis in this context. It is also common to average over the investigated models, weighted based on their relative plausibility, to infer model features (e.g., parameter values) and to make predictions.

#### Large datasets

Large datasets may constitute a computational bottleneck for model-based methods. It was, for example, pointed out that in some ABC-based analyses, part of the data have to be omitted [21]. A number of authors have argued that large datasets are not a practical limitation [10], [42], although the severity of this issue depends strongly on the characteristics of the models. Several aspects of a modeling problem can contribute to the computational complexity, such as the sample size, number of observed variables or features, time or spatial resolution, etc. However, with increasing computing power, this issue will potentially be less important.

Instead of sampling parameters for each simulation from the prior, it has been proposed alternatively to combine the Metropolis-Hastings algorithm with ABC, which was reported to result in a higher acceptance rate than for plain ABC [33]. Naturally, such an approach inherits the general burdens of MCMC methods, such as the difficulty to assess convergence, correlation among the samples from the posterior [23], and relatively poor parallelizability [10].

Likewise, the ideas of sequential Monte Carlo (SMC) and population Monte Carlo (PMC) methods have been adapted to the ABC setting [23], [45]. The general idea is to iteratively approach the posterior from the prior through a sequence of target distributions. An advantage of such methods, compared to ABC-MCMC, is that the samples from the resulting posterior are independent. In addition, with sequential methods the tolerance levels must not be specified prior to the analysis, but are adjusted adaptively [46].

It is relatively straightforward to parallelize a number of steps in ABC algorithms based on rejection sampling and sequential Monte Carlo methods. It has also been demonstrated that parallel algorithms may yield significant speedups for MCMC-based inference in phylogenetics [47], which may be a tractable approach also for ABC-based methods. Yet an adequate model for a complex system is very likely to require intensive computation irrespectively of the chosen method of inference, and it is up to the user to select a method that is suitable for the particular application in question.

#### Curse-of-dimensionality

High-dimensional datasets and high-dimensional parameter spaces can require an extremely large number of parameter points to be simulated in ABC-based studies to obtain a reasonable level of accuracy for the posterior inferences. In such situations, the computational cost is severely increased and may in the worst case render the computational analysis intractable. These are examples of well-known phenomena, which are usually referred to with the umbrella term curse-of-dimensionality [48].

To assess how severely the dimensionality of a dataset affects the analysis within the context of ABC, analytical formulas have been derived for the error of the ABC estimators as functions of the dimension of the summary statistics [49]–[50]. In addition, Blum and François have investigated how the dimension of the summary statistics is related to the mean squared error for different correction adjustments to the error of ABC estimators. It was also argued that dimension reduction techniques are useful to avoid the curse-of-dimensionality, due to a potentially lower dimensional underlying structure of summary statistics [49]. Motivated by minimizing the quadratic loss of ABC estimators, Fearnhead and Prangle have proposed a scheme to project (possibly high-dimensional) data into estimates of the parameter posterior means; these means, now having the same dimension as the parameters, are then used as summary statistics for ABC [50].

ABC can be used to infer problems in high-dimensional parameter spaces, although one should account for the possibility of overfitting (e.g., see the model selection methods in [39]–[40]). However, the probability of accepting the simulated values for the parameters under a given tolerance with the ABC rejection algorithm typically decreases exponentially with increasing dimensionality of the parameter space (due to the global acceptance criterion) [11]. Although no computational method (based on ABC or not) seems to be able to break the curse-of-dimensionality, methods have recently been developed to handle high-dimensional parameter spaces under certain assumptions (e.g., based on polynomial approximation on sparse grids [51], which could potentially heavily reduce the simulation times for ABC). However, the applicability of such methods is problem dependent, and the difficulty of exploring parameter spaces should in general not be underestimated. For example, the introduction of deterministic global parameter estimation led to reports that the global optima obtained in several previous studies of low-dimensional problems were incorrect [52]. For certain problems, it might therefore be difficult to know whether the model is incorrect or, as discussed above, whether the explored region of the parameter space is inappropriate [21]. A more pragmatic approach is to cut the scope of the problem through model reduction [11].

## Software for Application of ABC

A number of software packages are currently available for application of ABC to particular classes of statistical models. An assortment of ABC-based software is presented in Table 3.

**Table 3:** Software incorporating ABC.

Software	Keywords and Features	Reference
DIY-ABC	Software for fit of genetic data to complex situations. Comparison of competing models. Parameter estimation. Computation of bias and precision measures for a given model and known parameters values.	[53]
ABC R package	Several ABC algorithms for performing parameter estimation and model selection. Nonlinear heteroscedastic regression methods for ABC. Cross-validation tool.	[54]
ABC-SysBio	Python package. Parameter inference and model selection for dynamical systems. Combines ABC rejection sampler, ABC SMC for parameter inference, and ABC SMC for model selection. Compatible with models written in Systems Biology Markup Language (SBML). Deterministic and stochastic models.	[55]
ABCtoolbox	Open source programs for various ABC algorithms including rejection sampling, MCMC without likelihood, a particle-based sampler, and ABC-GLM. Compatibility with most simulation and summary statistics computation programs.	[56]
msBayes	Open source software package consisting of several C and R programs that are run with a Perl “front-end.” Hierarchical coalescent models. Population genetic data from multiple co-distributed species.	[57]
PopABC	Software package for inference of the pattern of demographic divergence. Coalescent simulation. Bayesian model choice.	[58]
ONeSAMP	Web-based program to estimate the effective population size from a sample of microsatellite genotypes. Estimates of effective population size, together with 95% credible limits.	[59]
ABC4F	Software for estimation of F-statistics for dominant data.	[60]
2BAD	Two-event Bayesian ADmixture. Software allowing up to two independent admixture events with up to three parental populations. Estimation of several parameters (admixture, effective sizes, etc.). Comparison of pairs of admixture models.	[61]

The suitability of individual software packages is dependent on the specific application at hand, the computer system environment, and the algorithms required.

## Conclusion

In conclusion, ABC represents a class of well-founded and powerful methods for Bayesian statistical inference. However, reliable application of ABC requires additional caution to be considered, due to the approximations and biases introduced at the different stages of the approach. In its current incarnation, the ABC toolkit as a whole is best suited for inference about parameters or predictive inferences about observables in the presence of a single or few candidate model(s). How to make ABC practically feasible for problems involving large sets of models and/or high-dimensional target parameter spaces is currently largely an open issue. Since the computation of the likelihood function is bypassed, it can be tempting to attack high-dimensional problems using ABC, but inevitably this comes bundled with new challenges that investigators need to be aware of at each step of their analyses.

## Supporting Information

Text S1 Version history of the text file (XML).
(XML)

Text S2 Peer reviews and response to reviews. Human-readable versions of the reviews and authors' responses are available as comments on this article.
(XML)
